# Seroprevalence of sexually transmitted infections over 44 years – A cross-sectional study in Sweden

**DOI:** 10.1177/09564624241248874

**Published:** 2024-04-24

**Authors:** Nirina Andersson, Tim Waterboer, Elisabet Nylander, Annika Idahl

**Affiliations:** 1 Department of Public Health and Clinical Medicine, Dermatology and Venereology, 174459 Umeå University, Umeå, Sweden; 2 Infections and Cancer Epidemiology, 28333 German Cancer Research Center (Deutsches Krebsforschungszentrum [DKFZ]), Heidelberg, Germany; 3 Department of Clinical Sciences, Obstetrics and Gynecology, 367314 Umeå University, Umeå, Sweden

**Keywords:** Chlamydia (Chlamydia trachomatis), bacterial disease, HPV (Human papillomavirus), viral disease, HSV (Herpes simplex virus)

## Abstract

**Background:**

Sexually transmitted infections (STIs) may cause substantial individual suffering and a large economic burden for society. This study examined the seroprevalence of *Chlamydia trachomatis*, *Mycoplasma genitalium*, herpes simplex virus (HSV) types 1 and 2, and several human papillomaviruses (HPV) in the Swedish population over time.

**Methods:**

The study population consisted of 30-year-old women attending maternity care, and 50 year-old men and women attending health check-ups, from 1975 to 2018. Antibody status was determined by multiplex serology and quantified using median reporter fluorescence intensity (MFI).

**Results:**

A total of 891 samples were analysed (519 from 30-year-old women, 186 from 50 year-old women and 186 from 50 year-old men). Of these, 41.5% showed seropositivity for *Chlamydia trachomatis*, 16.7% for *Mycoplasma genitalium*, 70.5% for HSV-1, 14.9% for HSV-2, 13.2% for high-risk HPV, and 8.3% for low-risk HPV. Seropositivity for *Mycoplasma genitalium*, HSV-1 and especially *Chlamydia trachomatis* decreased over time.

**Conclusions:**

There was a decrease over time in *Chlamydia trachomatis* seroprevalence, probably due to contact tracing, testing and early treatment; this might also have affected *Mycoplasma genitalium* seroprevalence. Despite the reduction, seroprevalences are still high, so continued and new efforts to reduce STI incidence are essential.

## Introduction

Sexually transmitted infections (STIs) are common and may cause severe symptoms and suffering for the individual^1,2^ as well as an economic burden for society.^3^ Some STIs are notifiable diseases regulated by the Communicable Diseases Act in Sweden, meaning that there is mandatory contact tracing and testing of all sexual partners. However, many infections cause no overt symptoms, despite conferring severe long-term sequelae,^4^ and so infected individuals may not seek health care and hence are not tested for STIs. Moreover, some STIs are not regulated by the Communicable Diseases Act, and so are not reported. This has led to a lack of knowledge on the incidence and prevalence of a range of STIs over time in the Swedish population; nevertheless, such data are important in the planning of preventive measures.

Infections with *Chlamydia trachomatis* (*C. trachomatis*), *Mycoplasma genitalium (M. genitalium),* human papillomavirus (HPV) and herpes simplex virus (HSV) are common diagnoses at Swedish STI clinics. *Neisseria gonorrhoea* *(N. gonorrhoea)* incidence has been low, with approximately three cases per 100,000 individuals every year during the 1990s.^5^

The most common bacterial STI in Sweden is *C. trachomatis*, with an incidence of 312 cases per 100,000 individuals in 2022.^6^ Since *C. trachomatis* was incorporated into the Communicable Diseases Act in Sweden in 1988, contact tracing has been mandatory. Nucleic acid amplification tests (NAAT) were introduced in the 1990s, increasing test sensitivity and specificity. In parallel to this, opportunistic screening has become widespread,^7^ meaning that testing is offered liberally to those in contact with healthcare for other reasons.

While NAAT for *M. genitalium* has been available since the 1990s,^8^ testing was uncommon until the 2000s and is still not recommended routinely due to increasing antibiotic resistance.^9^

HPV is a group of more than 200 types of viruses, some of which cause benign genital warts and others of which have cancer-causing potential.^10,11^ Vaccination against HPV was introduced in the Swedish general vaccination program for girls in 2010, and since 2020 has been offered to all children aged 10–11, regardless of gender. Both of the first two vaccines that were introduced protected against HPV types 16 and 18, which cause around 70% of all cervical cancer cases, and one of them also protected against HPV types 6 and 11, which cause 90% of all cases of genital warts.^6^ In addition to these four HPV types, the vaccine that has been used since 2019 also protects against types 31, 33, 45, 52 and 58, thereby covering around 90% of all cervical cancer cases.^12^

HSV type 1 infection typically affects facial nerves, causing oral lesions, but is nowadays as common as type 2 in genital infections. HSV infection may be reactivated and cause recurrent outbreaks of painful oral or genital blisters.^13^

Swedish maternal healthcare was implemented in the 1930s and developed over the following decades. One of the areas included in the program is the prevention of STIs. Since there is no national regulation on how this should be organized, different regions have different arrangements. Midwifery clinics offer testing for STIs in connection with contraceptive advice or maternity health care. All pregnant women are offered testing for HIV, hepatitis B and syphilis while testing for *C. trachomatis* and *N. gonorrhoea* have been offered liberally to those experiencing symptoms or who are in high-risk groups.^14^ Recently published guidelines however recommend that all pregnant women should be tested for *C. trachomatis* and *N. gonorrhoea*.^15^

In Sweden, statistics for the current incidence of STIs, such as *C. trachomatis*, *N. gonorrhoea* and syphilis are available since they are covered by the Communicable Diseases Act. However, a large percentage of, for example, *C. trachomatis* infections, are probably never diagnosed due to lack of clear symptoms. There is very little information on the occurrence of *M. genitalium*, HPV and HSV, since these infections are not regulated by the Communicable Diseases Act. As sexual habits change over time,^16^ and data on many infections is lacking, it is important to map the prevalence of STIs in different populations over time, in addition to those tested due to symptoms or contact tracing. This knowledge is important in the planning of preventive measures to reduce the incidence of STIs.

This study aimed to evaluate the cumulative incidence of several STIs (*C. trachomatis*, *M. genitalium*, HSV types 1 and 2, and several types of HPV), in the Swedish population over time, by examining their seroprevalence.

## Methods

The study was approved by the Swedish Ethical Review Authority (ref: 2019-00,173).

### Study design

This yearly cross-sectional study on STI seroprevalence included 30- and 50-year-old women and 50-year-old men in northern Sweden from 1975 to 2018.

### Study population

All samples analysed were supplied by Biobank North in collaboration with the Biobank Research Unit at Umeå University. The Northern Sweden Maternity Cohort (NSMC) was established in 1975 with the purpose of preserving serum samples for research. The cohort, which has been described previously,^17^ contains samples from women who have attended a maternity health care clinic in northern Sweden, mainly during the first trimester of pregnancy. The Västerbotten Intervention Programme (VIP) was established in 1985 as a population-based strategy. As part of the programme, blood samples and data are collected every year together with the Västerbotten health surveys, primarily from individuals aged 40, 50 and 60.^18^

This study included 12 randomly selected samples from every year between 1975 and 2018 for 30-year-old women from the NSMC and 12 samples (six female and six male) from every year between 1987 and 2017 for 50-year-old women and men from the VIP.

### Multiplex serology

Plasma samples were analysed at the German Cancer Research Center (DKFZ). Serostatus for antibodies was determined by multiplex serology as previously described.^19–22^ Briefly, this method uses recombinantly expressed and in situ affinity-purified viral and bacterial proteins as antigens in a glutathione S-transferase capture immunosorbent assay combined with fluorescent-bead technology. Sets of differently coloured beads, each carrying a different antigen, are mixed, and incubated with human sera, thus allowing parallel assessment of antibodies to up to 100 different antigens in a single reaction. Antibodies bound to the beads via the antigens are stained with anti-human-Ig and streptavidin-R-phycoerythrin. A Luminex analyser then identifies and quantifies each antigen based on internal bead colour and reporter fluorescence, respectively.

Both seroprevalence and antibody relativities were measured. The quantity of bound antibodies was determined as the median reporter fluorescence intensity (MFI) of at least 100 beads per antigen per specimen. Determination of seropositivity for the specific antibodies was based on MFI. Laboratory staff blinded to the status of the participants performed the testing. This serological methodology has been applied previously in EPIC studies on the association of HPV with head and neck cancer,^23^ cervical cancer^24^ and anogenital cancer.^25^ Antibodies for *C. trachomatis*, *M. genitalium*, HPV (types 6, 11, 16, 18, 31, 33, 45, 52 and 58) and HSV (types 1 and 2) were analysed. Antigen-specific cut-off values are presented in Table 1. Seropositivity for *C. trachomatis* was defined as either positive pGP3 or three positives out of momp-D, momp-A, momp-L2, tarp-Cter, tarp-Nter and HSP 60-1.^21,22^ Due to high cross-reactivity between momp serovars, and low prevalence of trachoma (serovar A-C) and Lymphogranuloma venereum (L1-3) in Sweden, momps representing all three biovars were included. For *M. genitalium*, seropositivity was defined as both MgPa Nter and rMgPa being positive. *N. gonorrhoea* serology is inherently difficult due to several reasons: 1) there is a high and rapid antigenic variation of *N. gonorrhoea*; 2) in many cases the immune response is not strong enough to elicit detectable levels of antibodies in the bloodstream; 3) some antibodies produced in response to *N. gonorrhoea* may cross-react with other *Neisseria* species that are part of the normal flora in the human body.^26^ Because of these challenges, and the very low prevalence expected in the study population, *N. gonorrhoea* serostatus was not assessed in this study.

**Table 1.** Cut-off median fluorescence intensity (MFI) values, analyzed using multiplex fluorescent bead-based serology at a dilution of 1:100.

Human papillomavirus (HPV)	6L1	571
	11L1	500
	16L1	422
	16 × 10^6^	484
	18L1	394
	31L1	712
	33L1	515
	45L1	368
	52L1	547
	58L1	371
Herpes simplex virus (HSV) 1	gG	200
HSV 2	mgG	300
*Chlamydia trachomatis*	pGP3	500
	momp-D	300
	momp-A	300
	momp-L2	300
	tarp-Cter	300
	tarp-Nter	300
	HSP 60-1	300
*Mycoplasma genitalium*	MgPa Nter	500
	rMgPa	500

### Data analysis and statistics

For interpretation of the results, samples were grouped by decade (Table 2). HPV types were divided into low-risk (types 6 or 11) or high-risk (types 16, 18, 31, 33, 45, 52, and 58) based on oncogenic potential.^27^ Seropositivity prevalences were calculated in version 28.0 of SPSS (IBM Corp). An overall comparison of change in seroprevalence over decades was performed using the linear-by-linear association test. For further exploration, pairwise comparisons of proportions between consecutive decades were made using the two-sample z-test. Graphs were generated using version 9.4.1 of GraphPad Prism.

**Table 2.** Number of samples in the study population, per decade.

	30 year-old women	50 year-old women	50 year-old men
1975–1979	52		
1980–1989	119	18	18
1990–1999	120	60	60
2000–2009	120	60	60
2010–2018	108	48	48

## Results

One sample from a 30 year-old woman in 1982 was excluded due to bead count below threshold, leaving a total of 891 samples for analysis. Seropositivity for all analysed STIs in the entire study population was 41.5% for *C. trachomatis*, 16.7% for *M. genitalium*, 70.5% for HSV-1, 14.9% for HSV-2, 13.2% for high-risk HPV and 8.3% for low-risk HPV.

**Figure 1.**
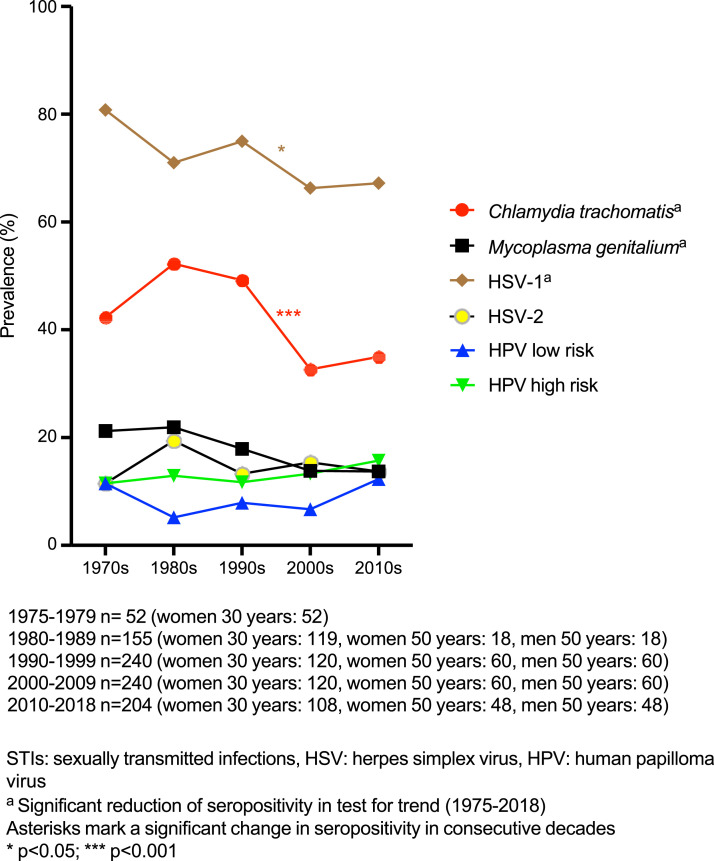
Seropositivity for STIs in the entire study population.

**Figure 2.**
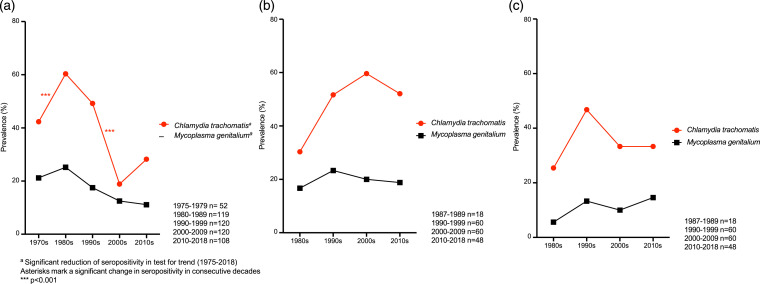
(a) Seropositivity for *Chlamydia trachomatis* and *Mycoplasma genitalium* among 30 year-old women. (b) Seropositivity for *Chlamydia trachomatis* and *Mycoplasma genitalium* among 50 year-old women. (c) Seropositivity for *Chlamydia trachomatis* and *Mycoplasma genitalium* among 50 year-old men.

**Figure 3.**
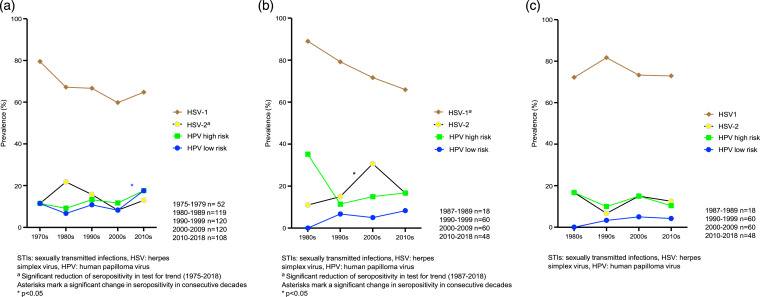
(a) Seropositivity for viral STIs among 30 year-old women, (b) Seropositivity for viral STIs among 50 year-old women, (c) Seropositivity for viral STIs among 50 year-old men.

Testing for trends in the entire study population revealed significant decrease over time in seropositivity for *C. trachomatis* (*p* < .001), *M. genitalium* (*p* = .015) and HSV-1 (*p* = .029, Figure 1). Testing for trends in subgroups showed significant decrease in seropositivity for *C. trachomatis* (*p* < .001, Figure 2(A)), *M. genitalium* (*p* = .003, Figure 2(A)) and HSV-2 (*p* = .047, Figure 3(A)) among 30-year-old women and for HSV-1 (*p* = .003, Figure 3(B)) among 50-year-old women. No other significant trends in seroprevalence were found among women, and none in men.

Comparison of consecutive decades in the entire study population revealed a decrease in HSV-1 seropositivity from 75.0% in the 1990s to 66.3% in the 2000s (*p* = .035, Figure 1) and a decrease in *C. trachomatis* seropositivity from 49.2% in the 1990s to 32.5% in the 2000s (*p* < .001, Figure 1). In subgroup analyses, seropositivity for *C. trachomatis* among 30-year-old women increased from 42.3% in the 1970s to 60.5% in the 1980s (*p* < .001), and then decreased from 49.2% in the 1990s to 18.3% in the 2000s (*p* < .001, Figure 2(A)). Low-risk HPV seropositivity among 30-year-old women increased from 8.3% in the 2000s to 17.6% in the 2010s (*p* = .036, Figure 3(A)). Among 50-year-old women, seropositivity for HSV-2 increased from 15.0% in the 1990s to 30.0% in the 2000s (*p* = .049, Figure 3(B)).

## Discussion

This is, to our knowledge, the first study describing the seroprevalence of multiple STIs over several decades in a sample representative of the general population in Sweden. Seropositivity for *C. trachomatis*, *M. genitalium* and HSV-1 decreased between 1975 and 2018. The decrease for *C. trachomatis* was most pronounced, especially from the 1990s to the 2000s, and especially among 30 year-old women. Among 50 year-old women on the other hand, there was an increase in *C. trachomatis* seropositivity from the 1980s to the 2000s. This increase was not statistically significant, possibly due to small sample size. The peak among 30 year-old women in the 1980s in combination with the peak among 50 year-old women in the 2000s suggests that the women who were 30 years old in the 1980s and 50 years old in the 2000s might represent a cohort with high-risk sexual behaviour. These seropositivity rates might seem high, but they match previously published data where a seropositivity of 46.3% was observed for *C. trachomatis* in Swedish women.^28^ The decrease in seropositivity for *C. trachomatis* is possibly an effect of mandatory contact tracing and quick and effective testing and treatment introduced in 1988.

In contrast to the decrease in seroprevalence seen in this study, previous research has shown that there was an increase in *C. trachomatis* diagnosis rate in Sweden between the 1980s and the 2000s; the diagnosis rate in 1990 was 311 cases per 100 000 population, compared to 391 cases per 100 000 in 2010.^6^ This increase could reflect a true increase in incidence but is more likely an effect of increased screening and partner tracing. We believe the discrepancy between the decrease in seroprevalence and increased diagnosis rate can in part be explained by the existence of quick and effective treatment that means an antibody response does not have time to form. One should also bear in mind that antibodies may persist for many years, and so a blood test at one point may reflect an infection several years earlier. A previous study showed that in two-thirds of cases, *C. trachomatis* momp antibodies persisted for less than 10 years after infection, but reinfection was shown to enhance antibody response.^29^ According to another study, pGP3 antibodies persisted for at least 12 years in 96% of cases.^30^

A possible reason for a decrease in *M. genitalium* prevalence could be intensified treatment for *C. trachomatis*, since doxycycline, which is the most common treatment for *C. trachomatis*, may cure 30%–40% of *M. genitalium*.^31–34^ Previous studies from the United States have shown increased condom use in the 1990s, partly due to fear of HIV acquisition^35^; this may also have contributed to the decreases in seropositivity seen in the present study.

Despite much preventive work and information provision, the incidence of STIs has steadily increased and is now at a relatively high level.^6^ Sexual habits have changed over time, for example, today’s students have less sexual experience and less non-penetrative sex than their counterpart from earlier decades.^16^ Moreover, there is no data regarding the incidence in populations other than those who have been tested. It is therefore of great importance to map the occurrence over time, in different populations, for use in preventive work.

Strengths of this study include the use of high-quality plasma samples over four decades, with sampling representative of the target population. The serology of several STIs was tested comprehensively, and we used pGP3 antibodies, which are currently the gold standard for *C. trachomatis* serology. However, the small sample size, in particular the population of 50-year-old women and men, is a limitation. The fact that the 30-year-old group consisted solely of pregnant women could be a possible source of bias, although it is difficult to know in which direction the results might be affected. One could speculate that pregnant women are more likely to be in committed relationships, with fewer sexual partners and less risk of acquiring STIs. However, as previously mentioned, antibodies may persist for many years and hence can reflect infections from years earlier.

In conclusion, we saw a pronounced decrease over time in seropositivity for *C. trachomatis*, probably due to contact tracing and quick and effective testing and treatment. As there has not been a corresponding decrease in *C. trachomatis* diagnosis rate, continued efforts to reduce STI incidence are essential.
